# How does heparin prevent the pH inactivation of cathepsin B? Allosteric mechanism elucidated by docking and molecular dynamics

**DOI:** 10.1186/1471-2164-11-S5-S5

**Published:** 2010-12-22

**Authors:** Mauricio GS Costa, Paulo R Batista, Cláudio S Shida, Charles H Robert, Paulo M Bisch, Pedro G Pascutti

**Affiliations:** 1 Instituto de Biofísica Carlos Chagas Filho, Universidade Federal do Rio de Janeiro, 21949-901, Rio de Janeiro, Brasil; 2 Universidade de Mogi das Cruzes, 08780-911, Mogi das Cruzes, Brasil; 3 CNRS Laboratoire de Biochimie Théorique, Institut de Biologie Physico Chimique, 75005 Paris, France

## Abstract

**Background:**

Cathepsin B (catB) is a promising target for anti-cancer drug design due to its implication in several steps of tumorigenesis. catB activity and inhibition are pH-dependent, making it difficult to identify efficient inhibitor candidates for clinical trials. In addition it is known that heparin binding stabilizes the enzyme in alkaline conditions. However, the molecular mechanism of stabilization is not well understood, indicating the need for more detailed structural and dynamic studies in order to clarify the influence of pH and heparin binding on catB stability.

**Results:**

Our pKa calculations of catB titratable residues revealed distinct protonation states under different pH conditions for six key residues, of which four lie in the crucial interdomain interface. This implies changes in the overall charge distribution at the catB surface, as revealed by calculation of the electrostatic potential. We identified two basic surface regions as possible heparin binding sites, which were confirmed by docking calculations. Molecular dynamics (MD) of both *apo* catB and catB-heparin complexes were performed using protonation states for catB residues corresponding to the relevant acidic or alkaline conditions. The MD of *apo* catB at pH 5.5 was very stable, and presented the highest number and occupancy of hydrogen bonds within the inter-domain interface. In contrast, under alkaline conditions the enzyme's overall flexibility was increased: interactions between active site residues were lost, helical content decreased, and domain separation was observed as well as high-amplitude motions of the occluding loop – a main target of drug design studies. Essential dynamics analysis revealed that heparin binding modulates large amplitude motions promoting rearrangement of contacts between catB domains, thus favoring the maintenance of helical content as well as active site stability.

**Conclusions:**

The results of our study contribute to unraveling the molecular events involved in catB inactivation in alkaline pH, highlighting the fact that protonation changes of few residues can alter the overall dynamics of an enzyme. Moreover, we propose an allosteric role for heparin in the regulation of catB stability in such a manner that the restriction of enzyme flexibility would allow the establishment of stronger contacts and thus the maintenance of overall structure.

## Background

Cathepsin B (EC 3.4.22.1) (catB) is one of the most well-characterized cysteine proteases, belonging to the clan CA (papain superfamily). In humans, its physiological role is implicated in bone resorption, antigen processing and protein turnover [1]. However, catB also participates in pathological processes such as cardiovascular disturbances [2], parasitic infections [3], Alzheimer's disease [4], osteoarthritis [5], tumor invasion and metastasis development [6,7]. Its main roles in cancer are *i)* its activity in directly cleaving extracellular matrix (ECM) components, *ii)* its activation of other ECM degrading proteases, which promotes tumor cell invasion into the surrounding tissue and bloodstream escape [8], and *iii)* stimulating angiogenesis which provides increased nutrients and oxygen supplies to cancer cells [9]. Thus, catB regulates several crucial steps in tumorigenesis and is a promising target for anti-cancer drug design [10].

**Figure 1:**
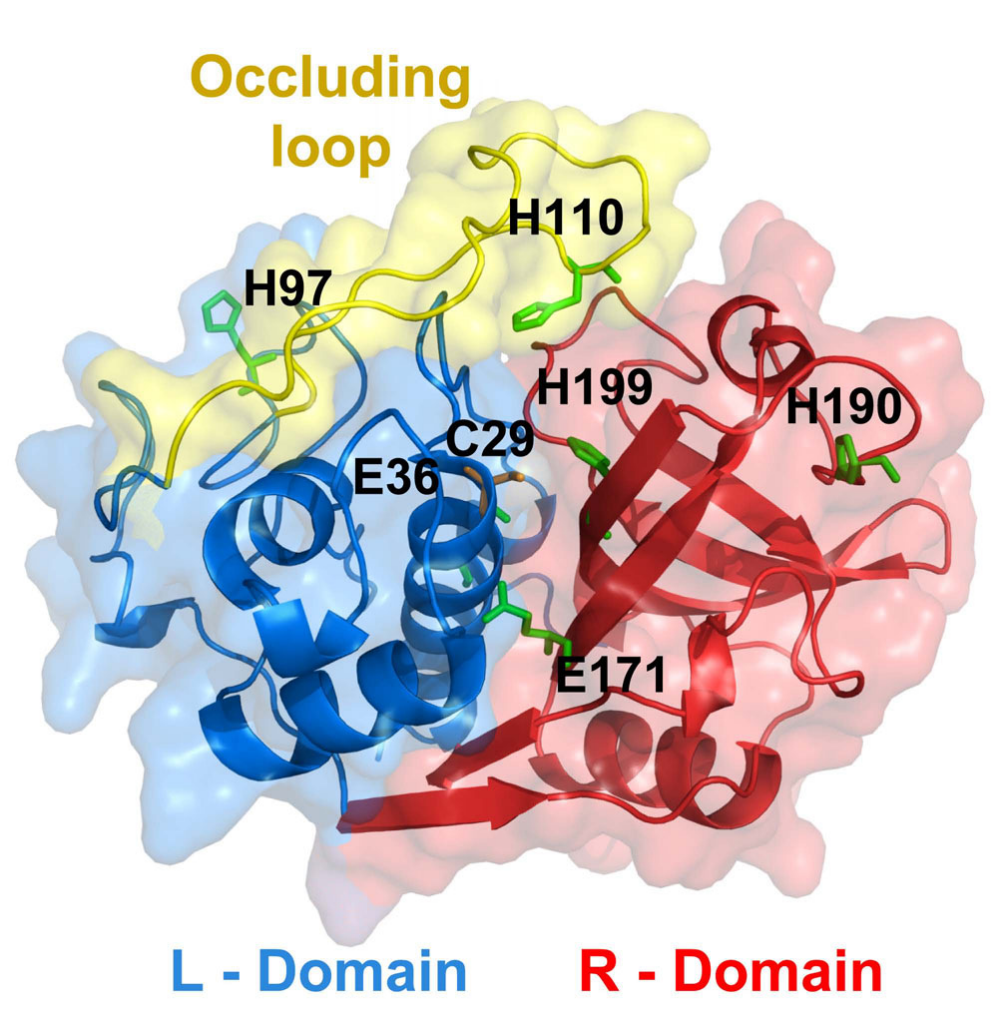
**Localization of differentially protonated residues in catB** Cartoon representation of the catB tertiary structure showing differentially protonated residues as green sticks. Protonation states were assigned to represent acidic (pH 5.5) or alkaline conditions (pH 8) based on pKa calculations with PROPKA on the catB crystal structure. The L and R domains of the protein are highlighted blue and red, respectively. The occluding loop (residues 106-124) a structural element found only in catB within its family, is represented in yellow.

Structurally, catB possesses the regular fold of papain-like enzymes, enclosing two distinct domains stabilized by six disulfide bridges, forming a large polar interface into which project the side chains of a few charged residues such as E171 and E36 (see Fig. 1). This interdomain interface is extremely important to catB overall activity as it comprises the active site residues (C29, H199 and N219). Unlike other members of the papain family, catB exhibits both exo- and endo-proteolytic activities. Its exo-activity is dependent on the presence of two adjacent histidine residues (H110 and H111) located at an insertion region called the “occluding loop”. These residues provide the necessary positive charge to anchor the negatively-charged C-terminal carboxylate of exo-substrates [11,12]. This region is only found in catB within its family, and it controls the access of large substrates to the active site [12]. Site-directed mutagenesis studies confirmed the role of the occluding loop since deletion of this entire region impairs exo- but not endo-proteolytic activity [13]. Additionally, this region confers thermal stability to catB and resistance against endogenous inhibitors such as cystatin C [13,14].

Currently the most potent and selective structure-based designed compounds available are derived from E-64 targeting the unusual occluding loop present in the catB 3D-structure [15]. However, enzymatic assays have shown that these inhibitors are strongly pH dependent as their optimal binding affinities are considerably diminished under neutral/alkaline conditions [16]. Since catB can be found in several cellular compartments with distinct pH values, these inhibitors are not effective *in vivo*. When catB is within the lysosomal or endosomal vesicles it confronts acidic conditions, in contrast to the neutral/alkaline environment when it is attached to membranes (mainly in caveolar microdomains) [17,18] or secreted in the ECM [19]. Although catB is rapidly inactivated under alkaline pH conditions, it was reported that membrane-associated forms are resistant to this process [20]. This peculiarity is believed to occur due to its interaction with heparan sulfate glycosaminoglycans (GAGs) on the cell surface [19]. This polysaccharide, structurally related to heparin, acts on the ECM as a co-receptor for several molecules such as growth factors and proteases [21] . Interaction between catB and heparin-like GAGs was already shown to prevent the enzyme's inactivation in high pH [22]. The main reported outcome was the maintenance of catB helical content in the presence of heparin at high pH, which was observed for papain as well [22,23]. Nevertheless, it has not been possible to precisely define the catB/heparin interaction sites and the molecular mechanism responsible for this protective effect.

Structural and molecular modelling studies can give insight into the molecular events concerning the modification of catB activity by pH changes and heparin binding. Some attempts have already been made towards designing new catB inhibitors using molecular modeling techniques [24,25], taking into account dynamical aspects of binding. However, these studies did not evaluate the influence of pH nor that of heparin binding on the modulation of the enzyme intrinsic flexibility.

In order to understand the heparin protective effect over catB structure we performed molecular dynamics (MD) simulations using an approach in which different pH conditions were taken into account by considering different protonation states of titratable groups on the protein surface. Further, docking analyses resulted in the identification of two heparin binding sites on catB structure. The MD calculations confirmed an increase of the overall catB flexibility and the loss of stability of the *apo* catB inter-domain interface in alkaline pH. The destabilization and the increased flexibility, notably in the occluding loop, were prevented by interaction with heparin, again in agreement with experimental data. We observed a role of active site residues in enzyme stabilization and in maintaining the helical content, and we propose an allosteric mechanism for the stabilizing effect promoted by GAG interaction. Taken together, our data provides an improved understanding of the molecular mechanisms responsible for both pH-induced inactivation and protection against inactivation by heparin binding.

## Results and discussion

### pH changes result in distinct protonation profiles

Prediction of pKa's for protein ionizable residues is an important tool for understanding features and catalytic mechanisms of pH-dependent enzymes [26]. We applied the PROPKA program to estimate pKa values in the catB structure and to determine the most probable corresponding protonation states of the enzyme under acidic and alkaline conditions. Two different conditions were studied: acidic (pH 5.5), and alkaline (pH 8.0), which allow comparison to available experimental data [22].

It is known that catalytic residues often present unusual pKa values compared to those of free amino acids in solution [27]. Accordingly, our results for catB predicted a pKa of 2.14 for the catalytic C29, in contrast to 8.0 expected in solution. Previous work on the papain catalytic cysteine (structurally related to C29 in catB) showed pKa values around 2.5 to 3.5 [28], in accordance to our estimation for C29.

Comparing the acidic and alkaline conditions we observed that six key residues are differentially protonated. Table 1 gives the pKa values for these residues and Fig. 1 shows their positions in the catB structure. We note that four of these residues belong to the interdomain interface (E36, H199, E171 and H110). The predicted pKa for E171 is 7.71, as carboxyl groups usually exhibit high pKa values in buried hydrophobic environments [26]. From our prediction we observed that both E171 and E36 are likely to be protonated at pH 5.5 but not in alkaline conditions.

**Table 1:** Differentially protonated residues in catB and pK_a_ values

		**Average values during 40ns MD**	

**Residue**	**predicted crystal**	**pH 5,5**	**pH 8**

**C29**	2,14	3.7 ± 1.5	**7.3 +1.6**

**E36**	5,9	5.4 ± 0.5	3.4 ± 1.2

**H97**	6,9	7.1 ± 0.2	7.3 ± 0.4

**H110**	6,6	6.6 ± 0.8	8.1 ± 0.7

**E171**	7,7	7.4 ± 0.2	6.5 ± 0.4

**H190**	7,2	7.1 ± 0.2	7.4 ± 0.7

**H199**	7,8	8.1 ± 2	**4.5 ± 1.7**

pK_a_ values predicted by PROPKA and the state of ionizable groups under different pH conditions are indicated. The protonation state of C29 at pH 8 were obtained taking into account deprotonation of H199 as previously proposed for papain. The protonation states of the catalytic residues (C29 and H199) are highlighted.

As expected, most of the residues with differing protonation states were histidines, with a pKa in solution of 6.5 and an ionization state that is very susceptible to pH changes in the physiological range. H110 is a key residue for stabilization of the occluding loop and is also crucial for the exo-proteolytic activity of catB, since along with H111 it anchors the carboxyl terminus of substrates. Interestingly, we observed an pKa of 7.79 for the catalytic H199, which implies that this residue is protonated only in acidic conditions. This result is correspondent to the measured pKa of H159 in papain [28]. In papain, it was previously reported that the ionization of the catalytic C25 and H159 residues are coupled [29]. There the deprotonation of H159 shifts the pKa of C25 from ~3.3 to 7.6, while the neutralization of C25 decreases the pKa of H159 from ~8.5 to 4.3. Due to the direct relationship between catB and papain we assigned the protonation states of these residues according to this proposed mechanism.

The pKa values predicted with PROPKA thus appear to be sensitive to local microenvironment changes around the ionizable residues. In order to verify if the applied protonation profiles accurately represent the distinct pH conditions simulated, we collected 1000 snapshots (one at each 400 ps) from each molecular dynamics (MD) trajectory in order to perform pKa calculations. Table 1 shows the average pKa values during the MD simulations. From both pH conditions we obtained values consistent with the protonation states predicted from the crystal structure, thus confirming their validity. In this Table we have also highlighted the pKa shift in the catalytic residues C29 and H199 as observed for papain.

### Electrostatic potential calculation and docking analyses reveal two heparin binding sites on catB structure

**Figure 2:**
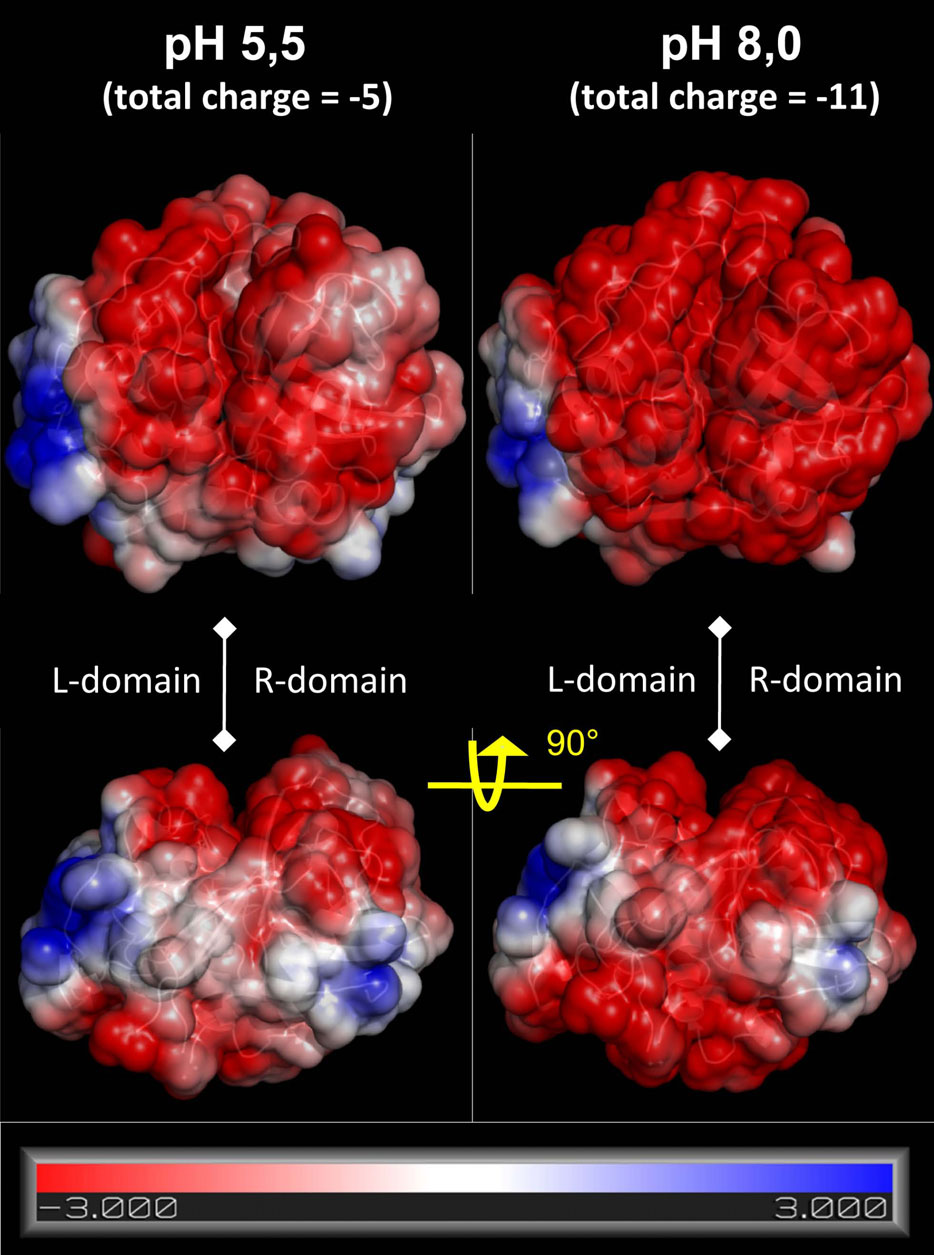
**Electrostatic surface of catB in distinct protonation states reveals two possible heparin binding sites** The electrostatic surface of catB using protonation states corresponding to acidic and alkaline pH conditions. Blue, red, and white indicate positively charged regions, negative areas and neutral regions, respectively, with the scale indicating ± k_b_T/e_c_, where k_b_ is the Boltzmann constant, T is the temperature and e_c_ is the charge of one electron. The heparin binding sites correspond to the basic regions localized in each domain.

The electrostatic potential at the catB surface revealed the influence of the different protonation states for each of the pH conditions considered. Overall, independent of the conditions, the catB surface is mostly negative, presenting one region of positive potential in each domain (Fig. 2). Under alkaline conditions the overall surface is qualitatively more negative. The positive sites are composed of R85, K86, K130, K141 and K144 in the L-domain, and the catB N-terminus together with K154 and R235 in the R-domain. The protein's total charge changes from -11 at pH 8 to -5 at pH 5.5, with six key residues protonated exclusively under acidic conditions.

**Figure 3:**
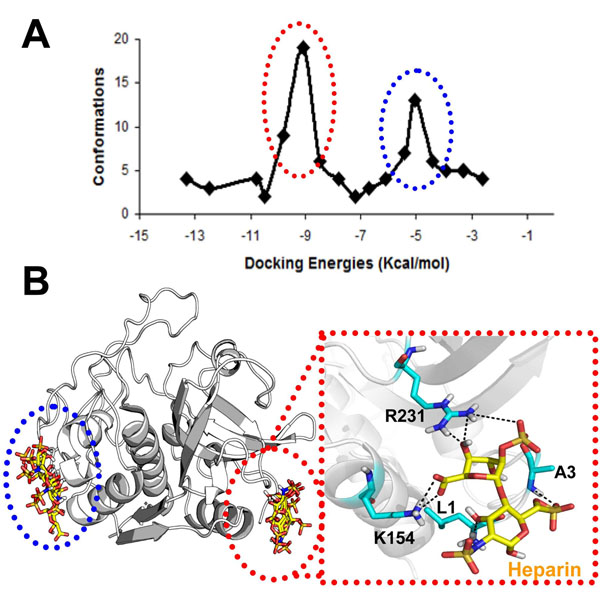
**Low-energy catB-heparin complexes** Docking calculations were performed in two steps: firstly a blind docking calculation with grid spacing of 0.498Å, which revealed the two positive sites in catB surface as preferential heparin binding sites. Subsequently a more precise calculation were performed using a grid (spacing of 0.202 Å) centered in each site. A) Clustering analysis of 100 low-energy docking results for the first run using a 2 Å RMS deviation clustering criteria. B) Visualization of the low energy complexes obtained after the second run for each site. Inset: Enlarged view of the lowest-energy complex selected for MD simulations.

Experimental studies have provided knowledge about the affinity and kinetics of the catB-heparin interaction [22]. However, the precise binding site(s) has(have) not been defined. Taking into account that heparin-protein interactions are mainly driven by charge interactions due to the high number of sulfate groups found in the polysaccharide, we visually identified two regions of positive potential at the catB surface as potential heparin binding sites. Blind docking calculations confirmed this prediction, as we found two low energy clusters of docking poses coincident with these positive regions (Fig. 3A and B). Almeida and coworkers proposed that heparin mainly interacts with the occluding loop [22]. From a structural view this hypothesis appears unlikely due to the lack of charge and shape complementarity in this region of the enzyme, and is consistent with the absence of low-energy clusters for such a mode of binding in the docking results. Moreover, we carried out another docking calculation verifying the atomic interactions in the heparin binding sites more precisely (see Methods). In the lowest energy complex obtained, heparin interacts with catB in the R-domain with an energy of -11 Kcal/mol (Fig. 3B). Although the positive potential at this site is smaller than that found in the L-domain, in R-domain site the van der Waals interactions were more favorable (contributed mainly by L1 at the N-terminus, A3, K154 and R231). We suggest that for a disaccharide the R-domain site is more important for this interaction; for longer sugar chains the L-domain could be also important for proper heparin accommodation.

### catB protonation profile influences protein stability

**Figure 4:**
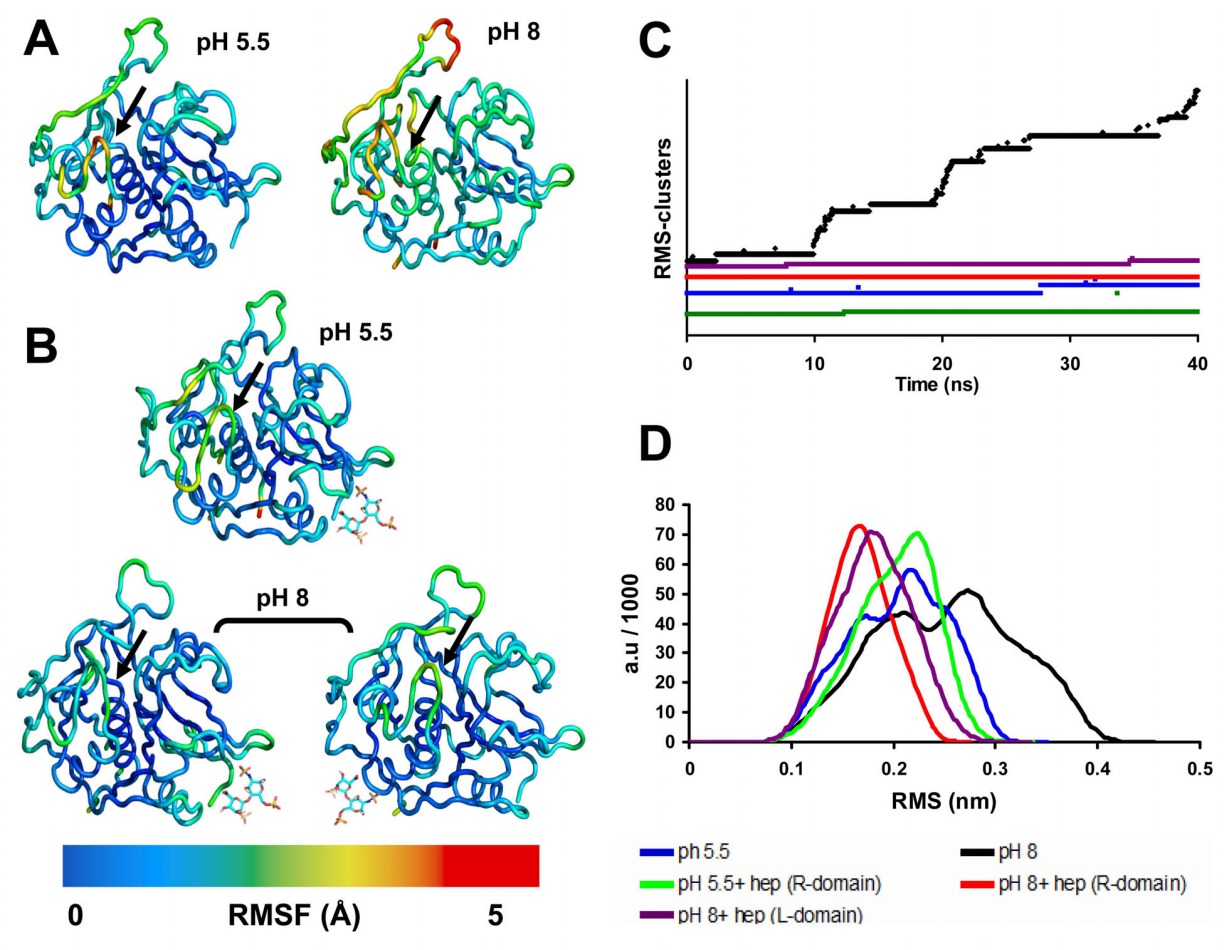
**Modification of catB flexibility at different pH conditions and upon heparin binding** A) Visualization of backbone flexibility colored according to residue RMS fluctuation values during the 40 ns MD of the *apo* form of catB in different pH conditions. The black arrows indicate the catalytic residue C29. B) The same as A but for the complexes catB-heparin. The disaccharide is represented by sticks and its position corresponds to the binding site (L or R) occupied. C) Time-evolution of RMSD clusters of catB backbone conformations (using a clustering criteria of 1 Å RMSD). Each system is colored differentially according to the legend D) Backbone RMSD distributions for each simulation. Colors definitions were applied as in C.

As flexibility plays a key role in protein biological functions [30], we analyzed the root mean square fluctuation (RMSF) of catB backbone residues during MD for each condition simulated. A direct influence of protonation state on the overall enzyme flexibility is clearly seen. Figure 4A shows that at pH 5.5 the fluctuations were around 2Å, in contrast to RMSF values up to 5Å observed at pH 8.0. This behavior was reproducible: we performed replicate simulations (40 ns each) for each condition using different initial conditions (see Methods), with equivalent results (Additional file 1). In addition, we observed that functional regions such as the occluding loop and the active site presented the highest fluctuations at pH 8.0, revealing that alkaline conditions rapidly affected catB flexibility in regions directly involved with activity, independent of the starting structure (Fig. 4A and Additional file 1). Remarkably, this result shows that changes in protonation of a few residues can alter both local and non-local properties (hydrogen bonds, hydration layers and polar contacts). Local perturbations due to changes in protonation states have already been observed via MD simulations (e.g. [31]). However, in our simulations both local and global flexibility (large-scale protein motions) of catB were modified as a function of pH, corroborating experimental findings where catB loses its activity in alkaline pH in parallel with large structural changes [22].

### Heparin binding prevents catB destabilization in alkaline pH

In MD simulations of the bound enzyme at pH 5.5, the RMS fluctuation profile was seen to be similar to that of the apo form (Fig. 4B). Hence, catB is stable under acidic conditions independent of heparin binding. In contrast, under alkaline conditions heparin-bound catB exhibited much smaller backbone fluctuations (2 Å) than the very flexible apo form in these conditions. Moreover, we observed similar results when simulations were started using different initial conformations and also with heparin docked at the other binding site (L domain) (Fig. 4B). Functional regions including the occluding loop and the active site were also stabilized, showing that heparin binding at the predicted sites promotes a global stabilizing effect.

In Fig. 4C, we show the time-evolution of the number of distinct clusters of catB backbone conformations throughout the different MD simulations. Here each cluster contains conformations within an RMSD of 1 Å of the cluster center. If during a simulation the system visits numerous clusters, the RMSD from the starting structure will be very high, indicating conformational instability in the simulation. In contrast, if a simulation visits only a few (correspondingly larger) clusters, it can be considered as being more conformationally stable. Indeed, we found few clusters during the simulations of *apo* catB and the catB-heparin complex (4 and 2 clusters, respectively) under protonation conditions corresponding to pH 5.5 (Fig. 4C). Furthermore, in acidic conditions the backbone RMSD distributions, similar to the RMS fluctuation profiles, did not change significantly on binding heparin, presenting deviations in both cases centered at 2.2 Å. However, in the heparin-bound simulation in acidic conditions, the RMSD distribution began to shift towards a conformational state far from the native structure. (Fig. 4D).

Considering that catB loses stability and activity in alkaline conditions [22], in the systems simulated under protonation conditions corresponding to high pH we expected to observe a higher number of backbone clusters. This expectation was confirmed: we observed 66 clusters in the *apo* catB simulation (Fig. 4C). In addition, under these conditions the catB structure does not stabilize during the simulation, with a continuous increase of backbone RMSD values throughout the 40 ns production period. A similar profile was observed in the replica simulation and also when starting with another conformation (a separate PDB structure, see Methods), in which we found 47 clusters. The RMS fluctuations of both replica simulations, plus the simulation performed with the alternative catB structure (Additional file 1), showed similar results to the *apo* enzyme in alkaline conditions (Fig. 4A).

The RMSD distribution plot shows a clear shift dividing into two populations: one around 2 Å and the other at higher RMSD values. (Fig. 4D). On the other hand, in the simulation with heparin under alkaline conditions we observed fewer clusters comparable to acidic conditions (Fig. 4C). This stabilization afforded by heparin binding is also reflected in the RMSD distribution presenting a narrowed profile centered at small RMSD values (around 1.7 Å) (Fig. 4D).

### Heparin prevents the domain separation observed in alkaline pH

**Figure 5:**
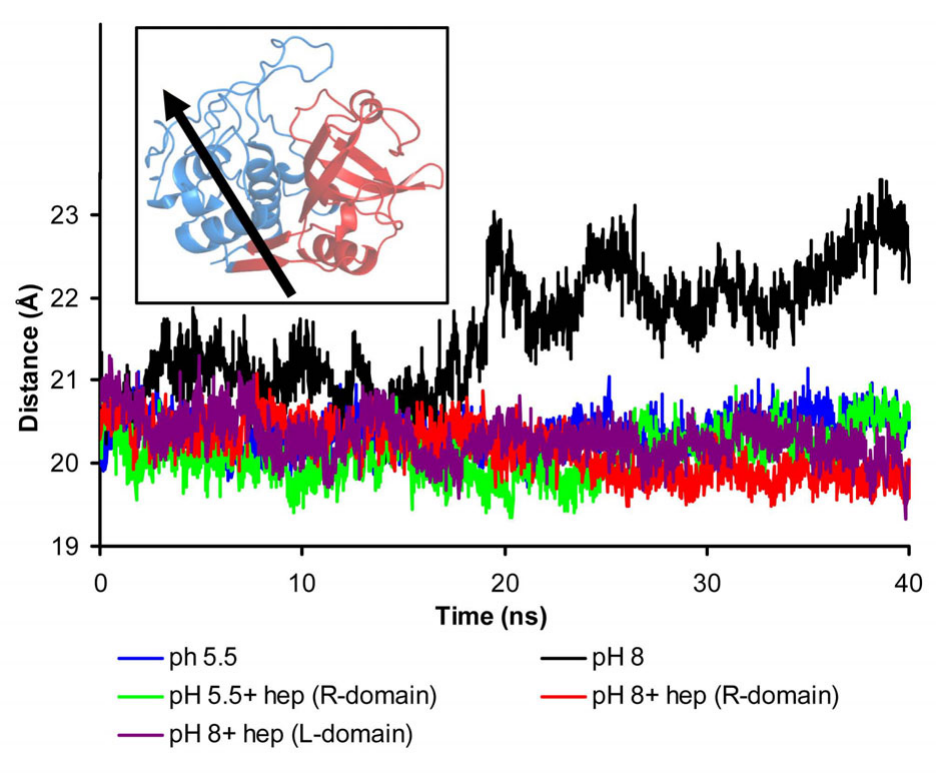
**Heparin prevents domain separation in alkaline pH** Time evolution of the distance between the centers-of-mass of each catB domain (L and R) during the MD. The domain nomenclature follows those adopted for papain. The L-domain corresponds to the amino terminus of catB (with exception of the first 10 residues) up to Y148 and the last four carboxy-terminal residues. The R-domain comprises the first 10 residues and the segment that extends from Y148 until the last four residues. Inset: Domain definitions of catB (colored red and blue respectively) were submitted to Domain Select analysis and the main axis of overall movement for *apo* catB (pH 8) showing the direction of the domain separation effect. We selected for this analysis the starting structure and the average structure of the 40ns MD simulation.

It was previously postulated that the loss of inter-domain interface contacts should be a crucial event in catB inactivation in alkaline pH [32]. Furthermore, the interface between L and R domains is essential not only for tertiary structure stabilization but also for the enzyme activity, as the catalytic triad is formed with residues from both domains [33]. To address whether this phenomenon is modulated by heparin, we compared the evolution of the distance between the centers of mass of the two domains during the simulations. In acidic conditions, this distance was stable at approximately 20 Å throughout the entire simulations, and was not significantly affected by heparin binding (Fig. 5). In contrast, when the enzyme was simulated under protonation conditions corresponding to alkaline medium, we observed a remarkable domain separation, with domain center-of-mass distances of up to 24 Å (Fig. 5). Further, we observed that this separation increased progressively throughout the simulation, suggesting that total L and R domain separation could occur at longer time-scales. This certainly would affect catB catalysis, since the active site is found inserted into a V-shaped cleft between the L and R domains. A more detailed analysis provided by use of the DynDom software [34] revealed the separation of the L-domain as the principal overall motion of the enzyme in this condition, thus reinforcing this conclusion (Fig. 5). This domain motion was observed in all simulations except those containing heparin, in which it was not possible to identify large domain motions with the DynDom program. Remarkably, in these last systems, the interdomain distance remained at approximately 21 Å in alkaline conditions, comparable to acidic conditions. This shows that heparin binding governs the inter-domain stability even under unfavorable alkaline conditions and would presumably also allow the maintenance of protein activity.

### Essential dynamics analysis reveals an allosteric role for heparin

The results shown above suggest that catB as a whole and in particular the critical domain interface and occluding loop are stabilized by heparin binding to a relatively distant site in the R domain. From basic thermodynamic principles, preferential binding of ligand to the native state will generally confer stability to a macromolecule in what has been described as an allosteric mechanism, without necessity for direct interaction between the ligand binding site and the active site residues, and has been extensively described in phenomenological terms [35]. The structural information available for catB, coupled with macromolecular simulations, provides a means for investigating details of such a mechanism directly.

We applied an essential dynamics analysis to examine the most relevant global macromolecular motions occurring during the simulations. Such analyses have been largely applied in the understanding of biological functions in proteins since they provide an evaluation of large-scale movements that are often related to domain motions and conformational changes [36]. We thus diagonalized the atomic positional covariance matrix to obtain the eigenvectors and corresponding eigenvalues. Selecting the first five (largest amplitude) principal components, we could recover around 60% of the total motions for *apo* catB and around 56% for heparin-bound catB. Our analysis focused on the systems simulated under alkaline conditions in order to address how heparin influences catB flexibility and prevents domain separation as shown above. We point out that certain analyses involving averaged quantities such as the essential dynamics and clustering approaches can be best interpreted for stable systems; the precise results for the *apo* catB under alkaline conditions will to some extent depend on the starting conformation. However, even under these conditions the overall results obtained were similar for replicated runs.

**Figure 6:**
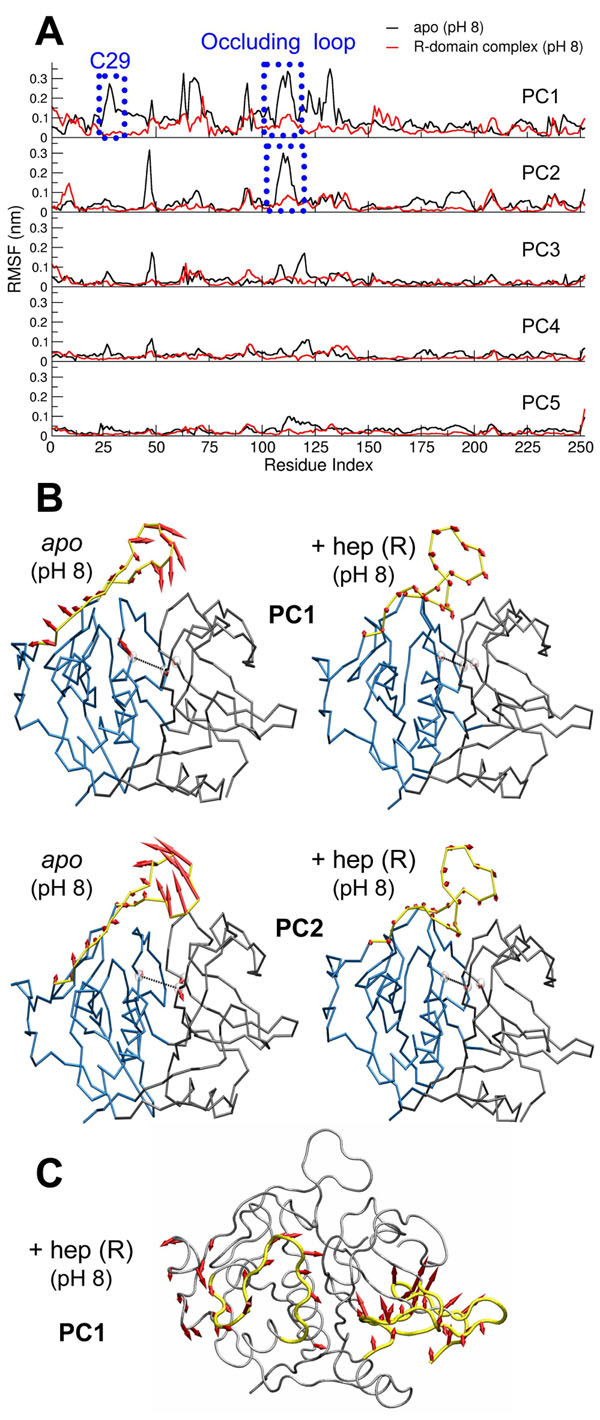
**Allosteric effect of heparin: stabilization of motions in functional regions of catB** A) RMS fluctuations of the trajectories in alkaline conditions calculated from projection of the MD trajectories onto the 5 principal components. B) Visualization of the motions of the active site and occluding loop provided by the first two principal components. The directions and amplitudes of the motions are represented by red arrows. C) Concerted motions in the R-domain complex. The regions with large amplitude movements are represented in yellow. The representation of directions and amplitudes is the same as B.

We examined the RMS fluctuations of each trajectory projected onto its five most representative principal components (PC). When comparing *apo* and complexed catB, we observed considerably lower fluctuations in the latter system for all PC analyzed (Fig. 6A). Further, we identified that the region around C29 and the occluding loop presented dramatically higher fluctuations in *apo* catB (reaching 3 Å in the two first PC), while in the complex these regions were shown to be very stable (deviations around 1Å in all PC). The movements corresponding to the first two components were seen to comprise relevant motions of both the occluding loop and active site. Figure 6B shows the directions of individual residue movements and also their amplitudes, which are proportional to the lengths of the arrows in this representation.

By inspecting other motions corresponding to the first component (PC1) in the R-domain complexes, we identified local changes in the binding region. The residues close to the polysaccharide (L1, A3 K156 and R233) adopted a new conformation. To verify that these motions would not lead to loss of binding interaction between catB and heparin, we measured the distance between the center of mass of heparin and the positive site during MD (Additional file 2) and found stable behavior: heparin remained bound during the entire simulation. Remarkably, however, this local motion is coupled to movements far from the heparin binding site. We observed that the residues 58 to 75 in the L-domain moved towards the interdomain interface (Fig. 6C). This motion certainly contributes to the stabilization of the overall structure, thus helping explain the results described above (Fig 4 and Fig 5). We did not find equivalent behavior in the L-domain complexes, which led us to investigate more deeply the correlated motions of catB in the *apo* form and in complex with heparin.

The analysis of cross correlation coefficients between pairs of residues is a useful method to identify correlated collective motions [37,38]. The correlation matrix represents the linear correlation between pairs of C-alpha atoms as they move about their average positions during dynamics. Positive correlations are related to motions occurring in the same direction whereas negative correlations indicate motion in opposite directions. We compared the correlation matrix of *apo* catB and of the complexes. In the *apo* form we observed positive intra-domain correlations but negative inter-domain correlations (Additional file 3). This pattern is not observed in the complexes, in which we identified a more diffuse pattern of correlations regardless of the binding site occupied. This result shows that heparin acts mainly as a stabilizing element, and its binding seems to restrict the anti-correlated collective motions that would be responsible for domain separation.

Concerning the occluding loop, we observed that the opening-closing movement is clearly represented by the two first components in *apo* catB. On the other hand, upon heparin binding these motions were not observed, independent of the binding site occupied. In addition, the first PC reveals the increasing distance between C29 and the other catalytic residues in *apo* catB, while in the complex this motion is absent. Therefore, heparin stabilizes catB motions in its functional regions. These results are consistent with experimental findings, which, although they revealed the implications of binding on the activity of the enzyme, did not provide a structural view of the phenomenon [22]. Heparin binding, despite the distance separating it from active site residues, prevents their disorganization as would otherwise be produced under alkaline conditions. The principal components obtained from the essential dynamics analysis of our MD simulations suggest that global concerted movements in the macromolecule leading to domain separation are suppressed by heparin binding, thus helping explain an allosteric role for heparin in this biological system.

### Heparin prevents loss of helical content and stabilizes the occluding loop

Loss of helical content was reported as the main effect related to alkaline-pH-induced inactivation of catB [22]. In the same study, it was reported that heparin prevented the loss of helical structure, which led to the postulate that the maintenance of activity in the catB-heparin complexes is strictly related to this structural aspect.

**Figure 7:**
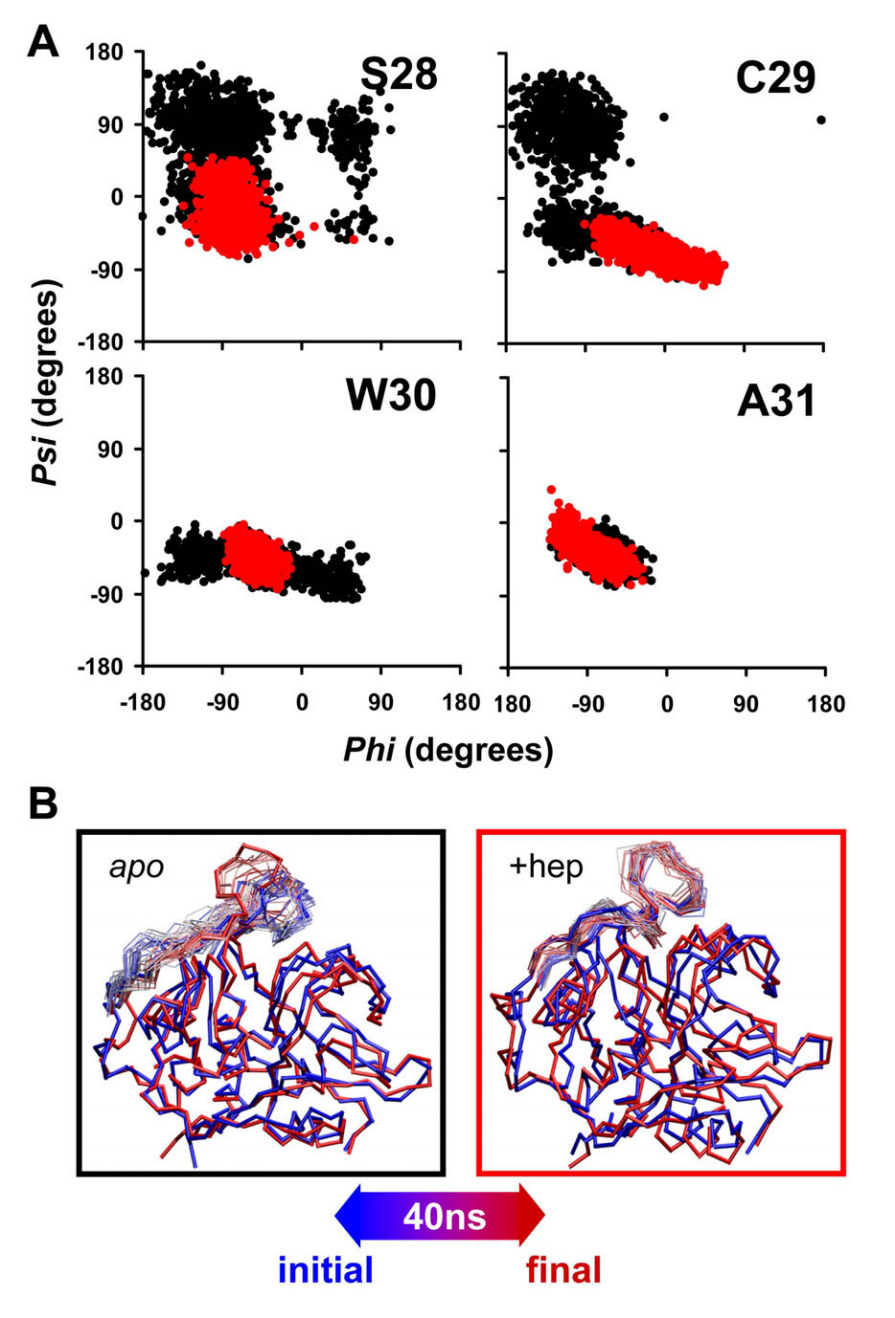
**Heparin binding stabilizes helical content in the active site and occluding loop** A) *Phi*-*Psi* distributions for the first residues of the main alpha helix (residues 28 to 45) in simulations with protonation corresponding to alkaline conditions. In black : apo catB; In red catB + heparin (R-domain). We present here the only the first four residues of the helix (S28, C29, W30 and A31) since the analysis on the subsequent residues did not shown observable differences in the distributions. B) Superposition of structural snapshots collected during MD, highlighting motions of the occluding loop. The color code from red to blue reflects the time evolution of the trajectory.

In analyzing the time evolution of secondary structural elements throughout the simulations, we observed stability of secondary structure elements in acidic conditions, independent of heparin binding (data not shown). In contrast, this same analysis for alkaline conditions revealed differences in the region of the main alpha helix (residues 28-45), which is located in the inter-domain interface. Figure 7A shows the *phi*-*psi* distribution plot for the first residues of the main helix in alkaline conditions. It is clear that heparin binding maintained this region in helical form under alkaline conditions, whereas in the *apo* simulations the same region assumed a random coil profile. The fact that heparin was bound far form this helical region again emphasizes an allosteric mechanism for this stabilization. We also believe that the loss of helical content seen here on the 40 ns timescale is just a precursor of a major loss of secondary structure at longer timescales.

It is discussed in the literature that papain-like enzymes, which possess a similar domain organization, present structural characteristics that confer some rigidity to the active site region [29]. The most important element supporting this characteristic is the network of interactions in the interdomain interface. We verified the stability of the interaction between catalytic residues C29-H199 and found that disorganization of the main alpha helix in apo catB under alkaline conditions is coupled with the separation of the catalytic residues (Additional file 5). These events are strongly correlated with the overall domain motions observed. The binding of heparin at a substantial distance form the interface nevertheless stabilizes the interdomain contacts maintaining the structuring of the active site, and would explain the results obtained in biochemical assays [22].

The occluding loop controls access to the active site and also confers the exo-proteolytic activity of the enzyme. In our simulations, we verified the open-close mechanism of this region and found that in acidic conditions a stable closed state is maintained regardless of heparin binding. However, at pH 8.0 the occluding loop exhibited high flexibility in the absence of heparin (Fig. 7B) as expected after RMSF analysis (Fig 4A). Nevertheless, under alkaline conditions, when heparin was bound the occluding loop exhibited a more stable conformation (Fig. 7B). In this conformation, besides closing the active site the occluding loop interacts with the R-domain in a distinct fashion from that observed in apo catB (Additional file 4). The occurrence of this conformation was also independent of the positive site occupied by heparin since the RMSD between the different complexes is around 1.4 Å. On the other hand, the loop conformations observed in the *apo* form simulations exhibit higher RMSD values (around 4.5 Å) compared to the complexes.

This result allows a discussion of experimental findings in which it was shown that heparin inhibits the exo-proteolytic activity of catB [22]. In that study, the authors proposed a competitive mechanism of inhibition due to the possible interaction of heparin with H110 and H111, which are the most important residues involved in accommodation of the negatively-charged C-terminal substrate carboxylate groups. From our analysis we conclude that the mechanism of inhibition promoted by heparin appears to be related also to the induction of conformational changes in catB, mainly in the occluding loop region, which adopts a distinct stable conformation that may impair proper binding of small substrates.

However, we cannot exclude the possibility that large heparin polymers may also bind to the occluding loop, thus inhibiting the exo-proteolytic activity of the enzyme, especially if the concentration of heparin increases as shown in ref. [22].

### A rearrangement of contacts explains heparin-induced stability at alkaline pH

The catB interface is mainly polar and is stabilized by ion pairs and hydrogen bonds between buried residues [12]. Since we see that pH changes induce distinct protonation profiles mainly at the interface, and also that heparin binding affects the interface stability, we analyzed the atomic interactions in this region. In particular, we verified the permanency of interactions between interfacial residues in order to see the influence of heparin binding on their stability (Table 2). We first checked certain interactions suggested by experimental studies, such as W30- E171. This interaction was observed only in the acidic pH simulations (87% apo catB / 85% catB-hep), and does not occur in alkaline pH (see Fig. 1 and Table 1) due to deprotonation of the E171 carboxyl group which interacts with the backbone of W30. The protonation state of E171 also affects the interaction between this residue and W80, since the observed occupancies were higher in acidic pH (88% apo catB / 87% catB-hep) than in alkaline conditions (37% apo catB / 68% catB-hep). This interaction was suggested to be important in the overall maintenance of the catB interface [12] and it was significantly stabilized by heparin binding. Other interactions found to be stabilized by heparin binding are detailed in Table 2.

**Table 2:** Occupancy of hydrogen bonds between interacting interface residues

Percentage of occupancy of hydrogen bonds between interface residues					
**Interaction**	**Type**	***apo* catB (pH 5,5)**	***Apo* catB (pH 8,0)**	**catB + hep (pH 5,5)**	**catB + hep (pH 8,0)**

**W30 – E171**	MC-SC	87%	**---**	85%	**---**

**W80 – E171**	SC-SC	88%	37%	87%	68%

**D81 – S150**	SC-SC	48%	6%	62%	55%

**R41 – P169**	SC-MC	70%	42%	83%	91%

**Q23 – S220**	MC-MC	65%	5%	82%	29%
	SC-SC	96%	**---**	90%	**---**

**E36-S220**	SC/SC	**---**	38%	**---**	72%

**D22-H110**	SC-SC	80%	6%	79%	67%

Interactions are broken down into main-chain (MC) and sidechain (SC). Domain definitions were used following papain domain organization.

**Figure 8:**
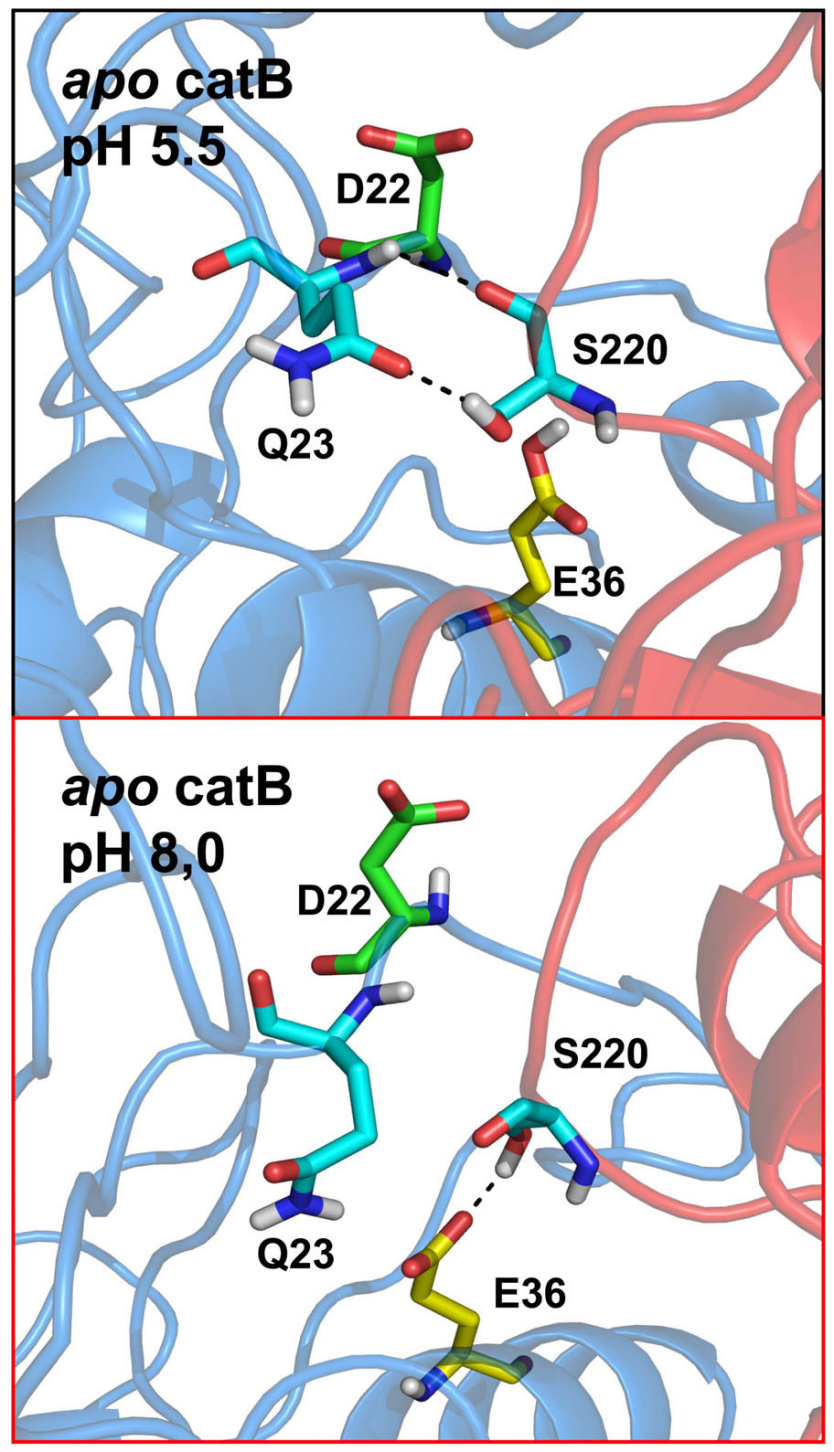
**Rearrangement of contacts explains the changes in flexibility of catB** View of the contacts established under the distinct pH conditions. In apo catB in acidic conditions two stable interactions are estabilished between Q23 and S220. Due to deprotonation of E36 in alkaline conditions, the pattern of interactions is changed and a new interaction is formed (E36 – S220). However, there is a loss of 1 hydrogen bond between !36 and S220 in this condition. Residues involved in the rearrangement are represented in stick representation and numbered. The conformation of D22 is represented since this residue is crucial in stabilization of the occluding loop.. Domains are colored blue and red (L and R, respectively). Hydrogen bonds are represented by dashed lines.

An important aspect observed through the H-bonding analysis was the network of contacts between Q23, E36 and S220 (Fig. 8). Concerning the Q23 – S220 contact, we observed high stability in acidic pH. We identified two stable H-bonds between these residues in acidic simulations, while in alkaline conditions only one weak interaction was found. Further, the occupancy for each of these contacts was dependent on heparin binding, since their prevalence increasing from 65% to 82% in acidic conditions and 6% to 29% at higher pH. It appears that the lack of one H-bond between Q23 and S220 in alkaline conditions is compensated by an interaction achieved between E36 and S220 only in such conditions. This occurs due to the distinctive protonation state of E36, which only when ionized (i.e., at pH 8) is able to interact with S220 through an H-bond. Remarkably, the occupancy of the E36-S220 interaction increases from 38% to 72% upon heparin binding. This result may be explained by the global stabilizing effect observed (see Essential dynamics analysis) which is crucial in the establishment of this distinct interdomain contact. The D22-H110 pair is another example of an interaction that was stabilized by the same mechanism. We measured the occupancy of this H-bonded state during the 40ns MD simulation. While in acidic pH conditions the occupancy of the hydrogen-bonded state was very high (80% apo catB / 79% catB-heparin), in alkaline conditions heparin binding significantly increased the stability of this interaction, which passed from 6% in *apo* catB to 67% in the catB-hep complex.

These results are correlated with the distinct loop conformation observed in the heparin bound complexes (Additional file 4). In addition, as shown, heparin binding at the R-domain induces a conformational change that leads to a more compact structure (Fig 6C). Further, heparin restricts collective motions involved in domain separation, which allows the stabilization of a distinct pattern of interactions formed in the interface under alkaline conditions.

## Conclusions

Heparin-protein interactions are known to regulate several biological processes including protease regulation [39], growth factor activity [40], macromolecular assembly [41] and viral mechanisms [42]. In this paper we unraveled the molecular mechanism of heparin protection against pH-induced inactivation of catB. This phenomenon was previously demonstrated through biochemical assays without a full understanding of the process because of the lack of structural information concerning heparin binding. Herein we were able to mimic the different pH conditions by performing MD simulations of catB with different protonation states according to pKa calculations. We confirmed experimental findings showing that under acidic conditions catB is stable. In contrast, at alkaline pH six residues are deprotonated, increasing the negative charge of the interdomain interface. This leads to charge repulsion and subsequent separation of the L and R domain. In addition, we observed the effect of the alkaline conditions in terms of destabilization of helical content, active-site disruption and increases in the flexibility of the occluding loop. All these events are closely related to loss of enzyme activity. Heparin binding counteracted these effects by a long-distance, allosteric mechanism which appears to be associated with *(i)* conformational changes leading to a more compact interface, and *(ii)* the restriction of catB flexibility, allowing stabilization and rearrangement of interdomain contacts under neutral/alkaline conditions. These new findings may be crucial for the design of new catB inhibitors.

## Methods

### Parameters for cathepsin B and heparin

The three-dimensional atomic coordinates of heparin were taken from the Protein Data Bank (entry 1HPN) [43]. From this structure we considered a heparin disaccharide for our studies. For docking and molecular dynamics we used the partial charges and force field parameters for heparin described in ref. [44]. The human cathepsin B crystal structure (PDB entry: 1HUC) [12] contains two proteins per asymmetric unit; the protein consisting of chains A and C was used as the starting structure for docking and MD simulations. The coordinates of catB complexed with CA-030 (PDB entry: 1CSB) [45] were also used to increase conformational sampling. We removed the coordinates of the inhibitor and the enzyme was treated as an *apo* form.

### pKa calculations

To obtain the pKa values for titratable catB residues we applied the PROPKA web server [26], using the catB crystal structure as input. This tool uses empirical parameters to estimate pKa values of each titratable residue in its chemical micro-environment, considering the contribution of H-bond formation, charge interactions and desolvation effects. This method was recently compared to other pKa predictive tools obtaining excellent results [46].

### Electrostatic surface analysis

With the results of the pKa analysis, we determine the most probable protonation state for each pH condition and added hydrogen atoms accordingly. Electrostatic surface calculations were performed using APBS software [47]. This analysis combines the solvent accessible surface (SAS) calculation with the values of the electrostatic potential for each atom at the surface. This latter calculation is obtained by the resolution of the linearized Poisson-Boltzmann equation to obtain the surface charge distribution. Images were generated using PyMol [48].

### Molecular docking

The heparin disaccharide was docked as a ligand with free rotation for all its torsion angles (sulfate, hydroxyl groups and glycosidic bonds). Docking calculations were performed using the Lamarckian Genetic Algorithm (LGA) implemented in Autodock 3.0 [49], a program extensively used to predict proteins/polysaccharide binding modes [50]. We applied a two-step protocol. Initially we performed blind docking with a grid of probe-atom interaction energies and the electrostatic potential generated covering the whole protein with a spacing of 0.498 Å. This step was necessary to obtain the putative heparin binding sites on catB structure. Subsequently, we carried out runs taking into consideration only the regions around the most populated low energy clusters obtained from the blind docking. In this step we used a grid spacing of 0.202 Å to evaluate more precisely the atomic interactions in the binding sites. In all, we performed a total of 100 docking runs using a population of 200 individuals. The solutions within 2 Å root mean square deviation of each other were grouped in the same cluster, which were then ranked according to their docking energy values. We selected docked structures of heparin from the lowest energy clusters obtained in our calculations to assemble two distinct catB-heparin complexes, in which heparin was bound to either one or the other of the two catB domains. We identify these complexes according to the domain occupied by the sugar (L or R) and used these structures as starting points for the energy minimization / MD protocols.

### Molecular dynamics simulation details

Molecular dynamics (MD) simulations, energy minimization and trajectory analyses were carried out with the GROMACS 3.1 package [51,52] using the GROMOS96 (G53a6) forcefield [53]. Different pH conditions were mimicked by changing the protonation states of six key residues accordingly to pKa calculations and experimental data as described above. Explicit SPC water molecules [54] were used in all simulations, in which a 14 Å layer of water molecules were added around the solute molecules within a cubic water box, using periodic boundary conditions. Counter ions were inserted for system neutralization. LINCS [55] and SETTLE [56] were applied to constrain solute and solvent bonds, respectively. Temperature and pressure were kept at 310 K and 1 atm respectively by the Berendsen approach [57]. Electrostatic interactions were calculated with the PME method [58], using non-bonded cutoffs of 1.0 nm for Coulomb and 1.2 nm for van der Waals. The MD integration timestep was 2fs.

A three-step energy minimization protocol was used to avoid artifacts in atomic trajectories due to conversion of potential into kinetic energies: firstly, we applied the steepest-descent algorithm: *i)* 5000 steps with solute heavy atom positions restrained to their initial positions using a harmonic constant of 1 kJ/mol.nm in each Cartesian direction, allowing unrestrained water and hydrogen movement; and *ii).* 5000 steps with all atoms free to move. Subsequently, the conjugate-gradients algorithm was applied for further energy minimization until an energy gradient fell to 42 KJ/mol.nm. A preliminary MD (1 ns) with heavy atom positions restrained was performed to achieve solvent equilibration and system heating to 310 K. In this step, initial velocities were generated for each simulation. A replica simulation was performed for each system using different initial velocities. Then we performed a 3 ns unrestrained equilibration MD for each system followed by a 40ns production MD.

In the simulations of the heparin-bound complexes, we verified the heparin-catB interaction stability by measuring the time-evolution of the distances between the centers of masses of the heparin and catB positive domains (Additional file 2). This control is important to guarantee that the effects observed result from the maintenance of a stable complex during the entire simulation.

### Essential dynamics analysis

We used the last 20 ns of the catB trajectories to obtain the covariance matrix of C-alpha atomic positions. In this step we applied the g_covar module of GROMACS package. Rotation and translation motions were removed prior to covariance matrix calculation by least-squares superposition. All analyses were performed with the g_anaeig module of GROMACS.

### Cross correlation analysis

We projected the MD trajectory onto the first principal component, corresponding to the largest eigenvector, of the covariance matrix in order to visualize the extreme structures and the major fluctuations of the correlated motions. The correlation matrix, a N×N array whose *i-j* entry *Corr_ij_* summarizes the correlation between the motions of atoms *i* and *j*, is obtained from the reduction and normalization of the covariance matrix.

### Domain motions analysis

Domain motions were analyzed with the Domain Select module of the DynDom software. This program compares two available structures of the same protein and deduces the rigid-body movement of one domain (the moving domain) relative to the other domain (the fixed domain) in the same way as the DynDom program. We selected the starting structure of each MD and the average structure of the respective simulation to be compared.

### Clustering analysis

We applied the g_cluster module of GROMACS package to calculate the RMS clusters of catB backbone conformations. Our clustering criteria was 1Å RMSD.

## Competing interests

The authors declare that they have no competing interests

## Authors' contributions

MGSC designed the study, carried out all simulations and drafted the manuscript. PRB designed and coordinated the study, worked on data analysis and drafted the manuscript. CSS performed preliminary molecular docking and dynamics studies. CHR participated in interpreting the study and drafted the manuscript. PMB designed and coordinated the study. PGP designed and coordinated the study.

## Supplementary Material

Additional file 1 **Similar RMS profiles of deviations are independent of the starting structure.** A) Visualization of backbone flexibility colored according to residue RMS fluctuation values during the 40 ns MD of the *apo* form of catB using the crystal structure with entry:1HUC . B) Same as A but using the crystal structure of catB entry 1CSB. Click here for file

Additional file 2 **Heparin binding is stable throughout all the simulations** Mean values of the distances between the centers of mass of each binding site and heparin. Deviations are colored according to the binding site. Click here for file

Additional file 3 **Cross correlation analysis of catB C-alpha atoms** A) Cross correlation matrix for the *apo* catB C-alpha atoms. B) Same as A but for the R-domain complex. C) Same as B but for the L-domain complex. Correlations close to 1 (colored in red) are related to motions in the same direction, whereas negative correlations are related to motions in opposite directions. In the upper left of each matrix are represented correlations with absolute values higher than 0.5. The domain organization of catB is represented close to each axis to clarify the interpretation of domain motions. Click here for file

Additional file 4 **Heparin binding induces a distinct conformation of the occluding loop** Structural alignment of the average structures obtained from the MD simulations in alkaline conditions. Color definitions are represented. We represented the occluding loop region in tubes to highlight the distinct conformation adopted by this structural element when catB binds heparin. Click here for file

Additional File 5 **Active site behavior during MD of apo catB at pH 5.5** Close-up view of the active-site region .The C-alpha of each catalytic residue (C29, H199 and N219) is represented as an orange sphere. Click here for file

Additional File 6 **Active site behavior during MD of apo catB at pH 8.0** Close-up view of the active-site region .The C-alpha of each catalytic residue (C29, H199 and N219) is represented as an orange sphere. Click here for file

Additional File 7 **Active site behavior during MD of catB-heparin complex at pH 5.5** Close-up view of the active-site region .The C-alpha of each catalytic residue (C29, H199 and N219) is represented as an orange sphere. Click here for file

Additional File 8 **Active site behavior during MD of catB-heparin complex at pH 8.0** Close-up view of the active-site region .The C-alpha of each catalytic residue (C29, H199 and N219) is represented as an orange sphere. Click here for file

## References

[B1] Chapman HA Riese RJ Shi GP Emerging roles for cysteine proteases in human biology Annual review of physiology 1997 59 63 88 9074757 10.1146/annurev.physiol.59.1.63

[B2] Lutgens SP Cleutjens KB Daemen MJ Heeneman S Cathepsin cysteine proteases in cardiovascular disease Faseb J 2007 21 12 3029 3041 17522380 10.1096/fj.06-7924com

[B3] McKerrow JH Development of cysteine protease inhibitors as chemotherapy for parasitic diseases: insights on safety, target validation, and mechanism of action International journal for parasitology 1999 29 6 833 837 10480720 10.1016/s0020-7519(99)00044-2

[B4] Haque A Banik NL Ray SK New insights into the roles of endolysosomal cathepsins in the pathogenesis of Alzheimer's disease: cathepsin inhibitors as potential therapeutics CNS & neurological disorders drug targets 2008 7 3 270 277 18673211 10.2174/187152708784936653

[B5] Martel-Pelletier J Cloutier JM Pelletier JP Cathepsin B and cysteine protease inhibitors in human osteoarthritis J Orthop Res 1990 8 3 336 344 2324852 10.1002/jor.1100080305

[B6] Gocheva V Joyce JA Cysteine cathepsins and the cutting edge of cancer invasion Cell cycle 2007 6 1 Georgetown, Tex 60 64 17245112 10.4161/cc.6.1.3669

[B7] Jedeszko C Sloane BF Cysteine cathepsins in human cancer Biological chemistry 2004 385 11 1017 1027 15576321 10.1515/BC.2004.132

[B8] Joyce JA Hanahan D Multiple roles for cysteine cathepsins in cancer Cell cycle 2004 3 12 Georgetown, Tex 1516 1619 15539953 10.4161/cc.3.12.1289

[B9] Kostoulas G Lang A Nagase H Baici A Stimulation of angiogenesis through cathepsin B inactivation of the tissue inhibitors of matrix metalloproteinases FEBS letters 1999 455 3 286 290 10437790 10.1016/s0014-5793(99)00897-2

[B10] Palermo C Joyce JA Cysteine cathepsin proteases as pharmacological targets in cancer Trends in pharmacological sciences 2008 29 1 22 28 18037508 10.1016/j.tips.2007.10.011

[B11] Krupa JC Hasnain S Nagler DK Menard R Mort JS S2' substrate specificity and the role of His110 and His111 in the exopeptidase activity of human cathepsin B The Biochemical journal 2002 361 Pt 3 613 619 11802791 10.1042/0264-6021:3610613 PMC1222344

[B12] Musil D Zucic D Turk D Engh RA Mayr I Huber R Popovic T Turk V Towatari T Katunuma N The refined 2.15 A X-ray crystal structure of human liver cathepsin B: the structural basis for its specificity The EMBO journal 1991 10 9 2321 2330 1868826 10.1002/j.1460-2075.1991.tb07771.x PMC452927

[B13] Illy C Quraishi O Wang J Purisima E Vernet T Mort JS Role of the occluding loop in cathepsin B activity The Journal of biological chemistry 1997 272 2 1197 1202 8995421 10.1074/jbc.272.2.1197

[B14] Nagler DK Storer AC Portaro FC Carmona E Juliano L Menard R Major increase in endopeptidase activity of human cathepsin B upon removal of occluding loop contacts Biochemistry 1997 36 41 12608 12615 9376367 10.1021/bi971264+

[B15] Frlan R Gobec S Inhibitors of cathepsin B Current medicinal chemistry 2006 13 19 2309 2327 16918357 10.2174/092986706777935122

[B16] Cathers BE Barrett C Palmer JT Rydzewski RM pH Dependence of inhibitors targeting the occluding loop of cathepsin B Bioorganic chemistry 2002 30 4 264 275 12392705 10.1016/s0045-2068(02)00009-3

[B17] Cavallo-Medved D Rudy D Blum G Bogyo M Caglic D Sloane BF Live-cell imaging demonstrates extracellular matrix degradation in association with active cathepsin B in caveolae of endothelial cells during tube formation Experimental cell research 2009 315 7 1234 1246 19331819 10.1016/j.yexcr.2009.01.021 PMC2677760

[B18] Cavallo-Medved D Sloane BF Cell-surface cathepsin B: understanding its functional significance Current topics in developmental biology 2003 54 313 341 12696754 10.1016/s0070-2153(03)54013-3

[B19] Mohamed MM Sloane BF Cysteine cathepsins: multifunctional enzymes in cancer Nature reviews 2006 6 10 764 775 16990854 10.1038/nrc1949

[B20] Sloane BF Rozhin J Lah TT Day NA Buck M Ryan RE Crissman JD Honn KV Tumor cathepsin B and its endogenous inhibitors in metastasis Advances in experimental medicine and biology 1988 233 259 268 10.1007/978-1-4899-5037-6_29 3066154

[B21] Coombe DR Kett WC Heparan sulfate-protein interactions: therapeutic potential through structure-function insights Cell Mol Life Sci 2005 62 4 410 424 15719168 10.1007/s00018-004-4293-7 PMC11924458

[B22] Almeida PC Nantes IL Chagas JR Rizzi CC Faljoni-Alario A Carmona E Juliano L Nader HB Tersariol IL Cathepsin B activity regulation. Heparin-like glycosaminogylcans protect human cathepsin B from alkaline pH-induced inactivation The Journal of biological chemistry 2001 276 2 944 951 11016923 10.1074/jbc.M003820200

[B23] Almeida PC Nantes IL Rizzi CC Judice WA Chagas JR Juliano L Nader HB Tersariol IL Cysteine proteinase activity regulation. A possible role of heparin and heparin-like glycosaminoglycans The Journal of biological chemistry 1999 274 43 30433 30438 10521421 10.1074/jbc.274.43.30433

[B24] Lim IT Meroueh SO Lee M Heeg MJ Mobashery S Strategy in inhibition of cathepsin B, a target in tumor invasion and metastasis Journal of the American Chemical Society 2004 126 33 10271 10277 15315439 10.1021/ja0489240

[B25] Zhou Z Wang Y Bryant SH Computational analysis of the cathepsin B inhibitors activities through LR-MMPBSA binding affinity calculation based on docked complex Journal of computational chemistry 2009 10.1002/jcc.21214 PMC2735608 19242965

[B26] Li H Robertson AD Jensen JH Very fast empirical prediction and rationalization of protein pKa values Proteins 2005 61 4 704 721 16231289 10.1002/prot.20660

[B27] Harris TK Turner GJ Structural basis of perturbed pKa values of catalytic groups in enzyme active sites IUBMB life 2002 53 2 85 98 12049200 10.1080/15216540211468

[B28] Pinitglang S Watts AB Patel M Reid JD Noble MA Gul S Bokth A Naeem A Patel H Thomas EW A classical enzyme active center motif lacks catalytic competence until modulated electrostatically Biochemistry 1997 36 33 9968 9982 9254592 10.1021/bi9705974

[B29] Dardenne LE Werneck AS de Oliveira Neto M Bisch PM Electrostatic properties in the catalytic site of papain: A possible regulatory mechanism for the reactivity of the ion pair Proteins 2003 52 2 236 253 12833547 10.1002/prot.10368

[B30] Huber R Bennett WS Jr. Functional significance of flexibility in proteins Biopolymers 1983 22 1 261 279 6673759 10.1002/bip.360220136

[B31] Varma S Chiu SW Jakobsson E The influence of amino acid protonation states on molecular dynamics simulations of the bacterial porin OmpF Biophysical journal 2006 90 1 112 123 16183883 10.1529/biophysj.105.059329 PMC1367011

[B32] Turk B Dolenc I Zerovnik E Turk D Gubensek F Turk V Human cathepsin B is a metastable enzyme stabilized by specific ionic interactions associated with the active site Biochemistry 1994 33 49 14800 14806 7993907 10.1021/bi00253a019

[B33] Turk B Turk V Turk D Structural and functional aspects of papain-like cysteine proteinases and their protein inhibitors Biological chemistry 1997 378 3-4 141 150 9165064

[B34] Hayward S Berendsen HJ Systematic analysis of domain motions in proteins from conformational change: new results on citrate synthase and T4 lysozyme Proteins 1998 30 2 144 154 9489922

[B35] Robert CH Colosimo A Gill SJ Allosteric formulation of thermal transitions in macromolecules, including effects of ligand binding and oligomerization Biopolymers 1989 28 10 1705 1729 2597726 10.1002/bip.360281006

[B36] Berendsen HJ Hayward S Collective protein dynamics in relation to function Current opinion in structural biology 2000 10 2 165 169 10753809 10.1016/s0959-440x(00)00061-0

[B37] de Oliveira CAF Guimaraes CRW Barreiro G de Alencastro RB Human cytomegalovirus protease: Why is the dimer required for catalytic activity? Journal of Chemical Theory and Computation 2007 3 1 278 288 10.1021/ct600175x 26627171

[B38] Hunenberger PH Mark AE Vangunsteren WF Fluctuation and Cross-Correlation Analysis of Protein Motions Observed in Nanosecond Molecular-Dynamics Simulations Journal of molecular biology 1995 252 4 492 503 7563068 10.1006/jmbi.1995.0514

[B39] Jin L Abrahams JP Skinner R Petitou M Pike RN Carrell RW The anticoagulant activation of antithrombin by heparin Proceedings of the National Academy of Sciences of the United States of America 1997 94 26 14683 14688 9405673 10.1073/pnas.94.26.14683 PMC25092

[B40] Mach H Volkin DB Burke CJ Middaugh CR Linhardt RJ Fromm JR Loganathan D Mattsson L Nature of the interaction of heparin with acidic fibroblast growth factor Biochemistry 1993 32 20 5480 5489 7684608 10.1021/bi00071a026

[B41] Chen Y Maguire T Hileman RE Fromm JR Esko JD Linhardt RJ Marks RM Dengue virus infectivity depends on envelope protein binding to target cell heparan sulfate Nature medicine 1997 3 8 866 871 9256277 10.1038/nm0897-866

[B42] Harrop HA Coombe DR Rider CC Heparin specifically inhibits binding of V3 loop antibodies to HIV-1 gp120, an effect potentiated by CD4 binding AIDS 1994 8 2 London, England 183 192 8043225 10.1097/00002030-199402000-00005

[B43] Mulloy B Forster MJ Jones C Davies DB N.m.r. and molecular-modelling studies of the solution conformation of heparin The Biochemical journal 1993 293 Pt 3 849 858 8352752 10.1042/bj2930849 PMC1134446

[B44] Verli H Guimaraes JA Molecular dynamics simulation of a decasaccharide fragment of heparin in aqueous solution Carbohydrate research 2004 339 2 281 290 14698886 10.1016/j.carres.2003.09.026

[B45] Turk D Podobnik M Popovic T Katunuma N Bode W Huber R Turk V Crystal structure of cathepsin B inhibited with CA030 at 2.0-A resolution: A basis for the design of specific epoxysuccinyl inhibitors Biochemistry 1995 34 14 4791 4797 7718586 10.1021/bi00014a037

[B46] Davies MN Toseland CP Moss DS Flower DR Benchmarking pK(a) prediction BMC biochemistry 2006 7 18 16749919 10.1186/1471-2091-7-18 PMC1513386

[B47] Baker NA Sept D Joseph S Holst MJ McCammon JA Electrostatics of nanosystems: application to microtubules and the ribosome Proceedings of the National Academy of Sciences of the United States of America 2001 98 18 10037 10041 11517324 10.1073/pnas.181342398 PMC56910

[B48] Delano WL The PyMOL Molecular Graphics System 1998

[B49] Morris GM Goodsell DS Halliday RS Huey R Hart WE Belew RK Olson AJ Automated docking using a Lamarckian genetic algorithm and an empirical binding free energy function Journal of computational chemistry 1998 19 14 1639 1662

[B50] Bitomsky W Wade RC Docking of Glycosaminoglycans to Heparin-Binding Proteins: Validation for aFGF, bFGF, and Antithrombin and Application to IL-8 Journal of the American Chemical Society 1999 121 13 3004 3013

[B51] Hess B Kutzner C Van Der Spoel D Lindahl E GROMACS 4: Algorithms for Highly Efficient, Load-Balanced, and Scalable Molecular Simulation Journal of chemical theory and computation 2008 4 2 435 10.1021/ct700301q 26620784

[B52] Van der Spoel D Lindahl E Hess B van Buuren AR Apol E Meulenhoff PJ Tieleman DP Sijbers aLTM Feenstra KA van Drunen R Berendsen H.J.C. Gromacs User Manual version 3.2 2004

[B53] Oostenbrink C Villa A Mark AE van Gunsteren WF A biomolecular force field based on the free enthalpy of hydration and solvation: the GROMOS force-field parameter sets 53A5 and 53A6 Journal of computational chemistry 2004 25 13 1656 1676 15264259 10.1002/jcc.20090

[B54] Berendsen HJC PJ Van Gusteren WF Hermans J “Intermolecular Forces” 1981

[B55] Hess B Bekker H Berendsen HJC Fraaije JGEM LINCS: A linear constraint solver for molecular simulations Journal of computational chemistry 1997 18 1463 1472

[B56] Miyamoto S Kollman PA Settle - an Analytical Version of the Shake and Rattle Algorithm for Rigid Water Models Journal of computational chemistry 1992 13 952 962

[B57] Berendsen HJC Postma JPM Vangunsteren WF Dinola A Haak JR Molecular-Dynamics with Coupling to an External Bath J Chem Phys 1984 81 3684 3690

[B58] Ulrich E Lalith P Max LB Tom D Hsing L Lee GP A smooth particle mesh Ewald method J Chem Phys 1995 103 8577 8593

