# Supplementary figures and images for: How does heparin prevent the pH inactivation of cathepsin B? Allosteric mechanism elucidated by docking and molecular dynamics
Source: BMC Genomics. 2010 Dec 22;11(Suppl 5):S5. doi: 10.1186/1471-2164-11-S5-S5 (PMC3045798; doi:10.1186/1471-2164-11-S5-S5)

PDB entry: 1HUC (pH 8)

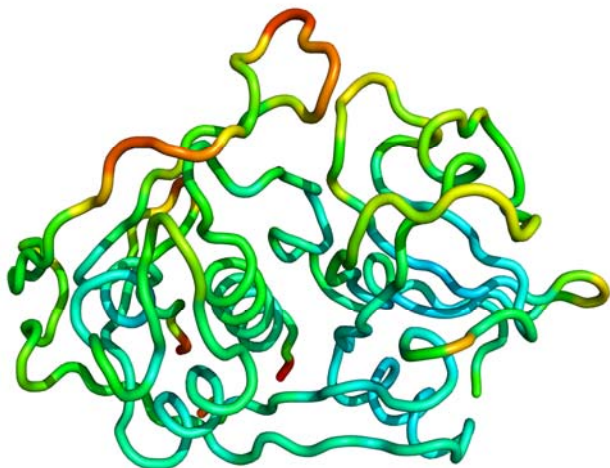

PDB entry: 1CSB (pH 8)

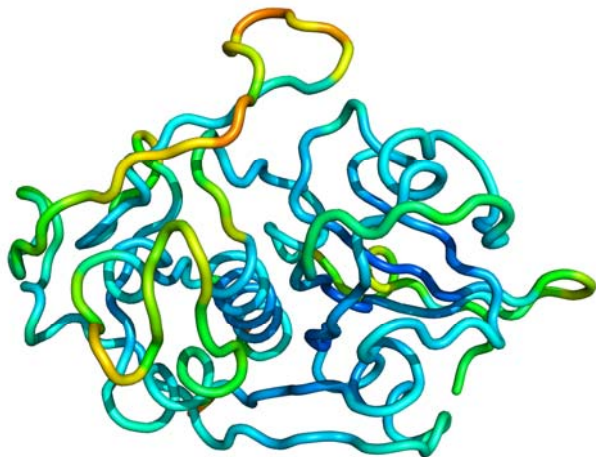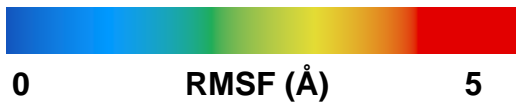

Supplement: Additional file 1 — Similar RMS profiles of deviations are independent of the starting structure. A) Visualization of backbone flexibility colored according to residue RMS fluctuation values during the 40 ns MD of the apo form of catB using the crystal structure with entry:1HUC . B) Same as A but using the crystal structure of catB entry 1CSB. [file 1471-2164-11-S5-S5-S1.pdf]

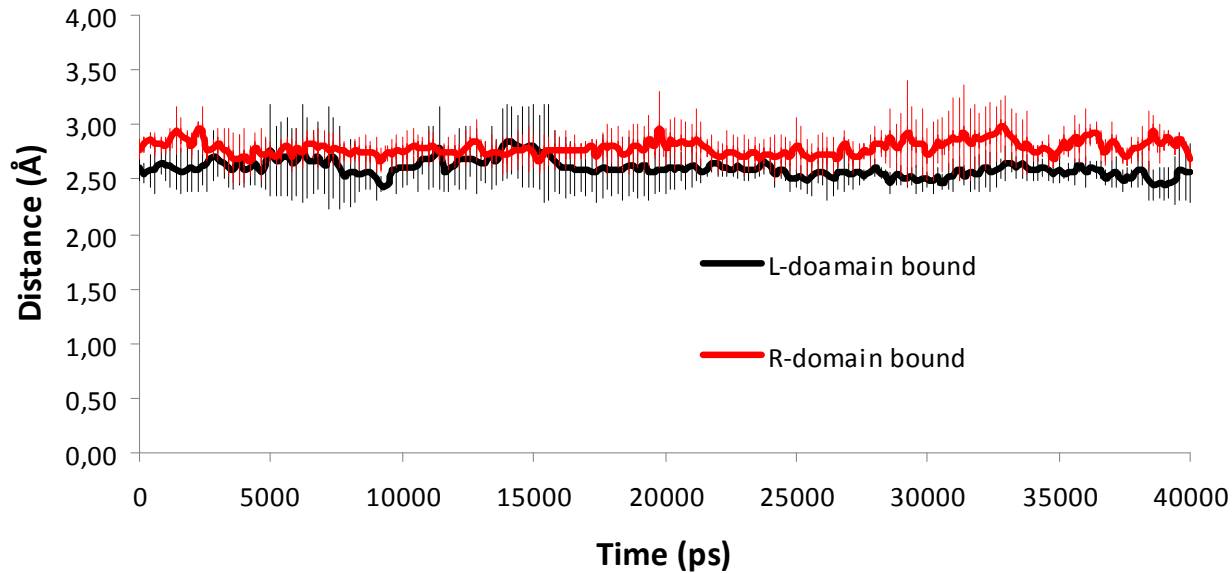

Supplement: Additional file 2 — Heparin binding is stable throughout all the simulations Mean values of the distances between the centers of mass of each binding site and heparin. Deviations are colored according to the binding site. [file 1471-2164-11-S5-S5-S2.pdf]

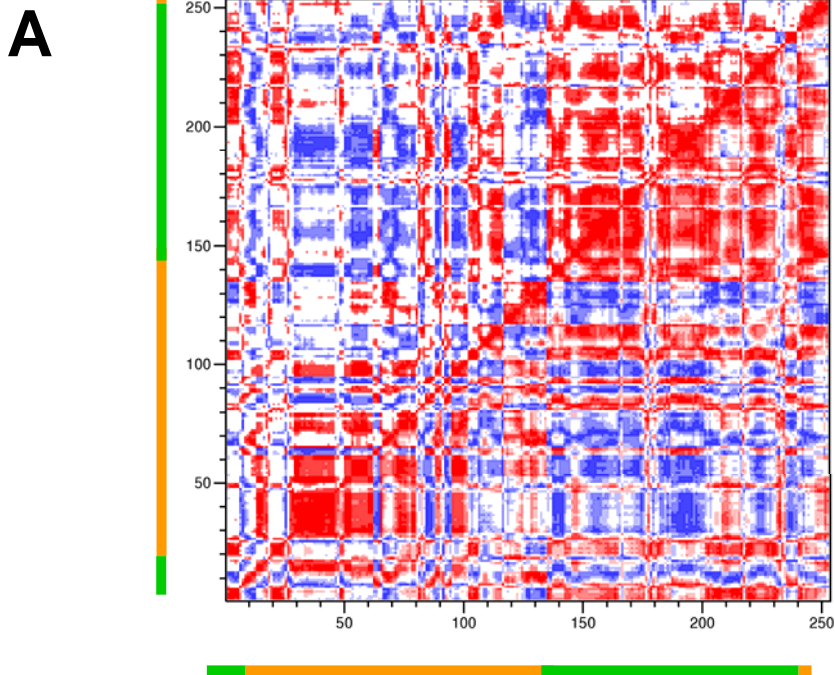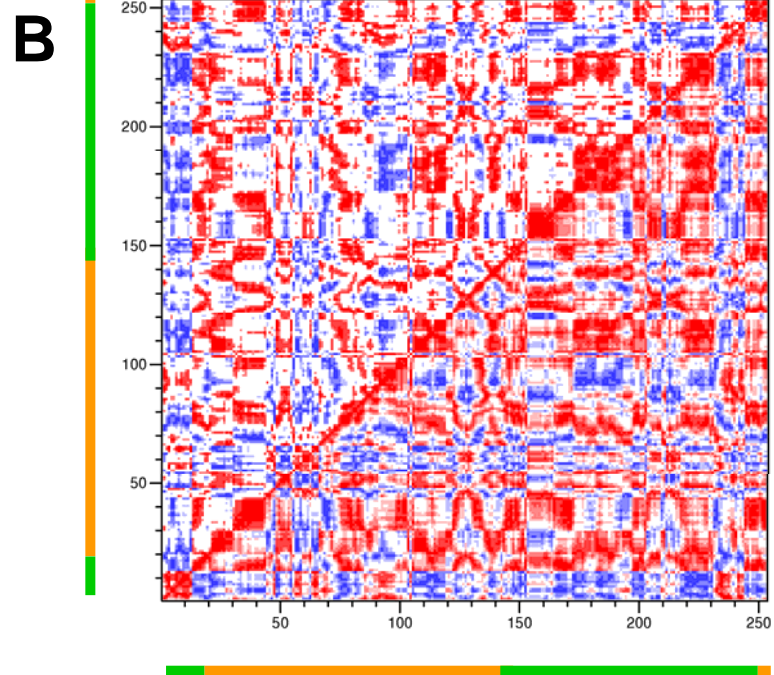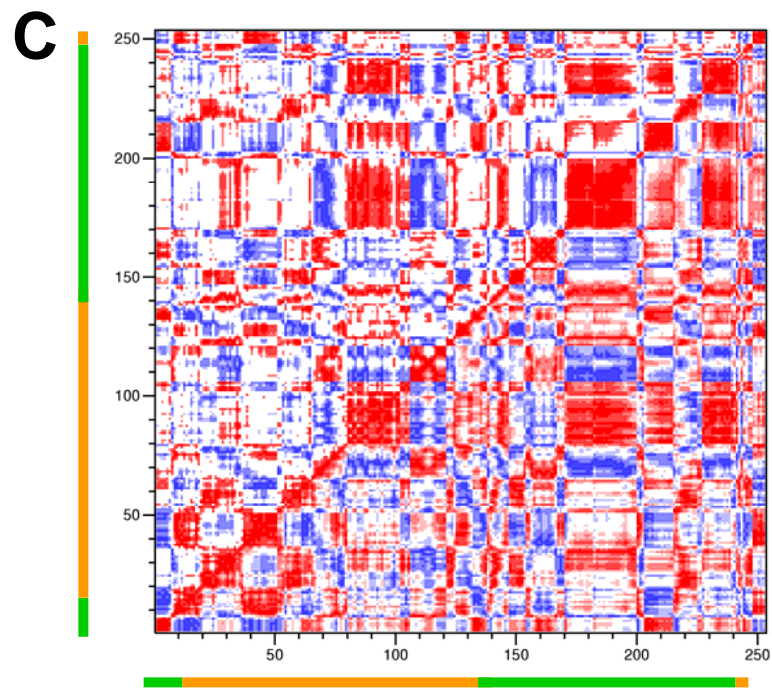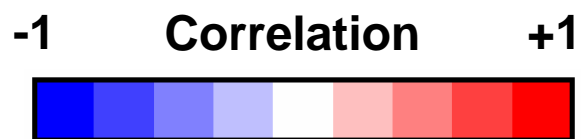

**L-domain** ———

**R-domain** ———

Supplement: Additional file 3 — Cross correlation analysis of catB C-alpha atoms A) Cross correlation matrix for the apo catB C-alpha atoms. B) Same as A but for the R-domain complex. C) Same as B but for the L-domain complex. Correlations close to 1 (colored in red) are related to motions in the same direction, whereas negative correlations are related to motions in opposite directions. In the upper left of each matrix are represented correlations with absolute values higher than 0.5. The domain organization of catB is represented close to each axis to clarify the interpretation of domain motions. [file 1471-2164-11-S5-S5-S3.pdf]

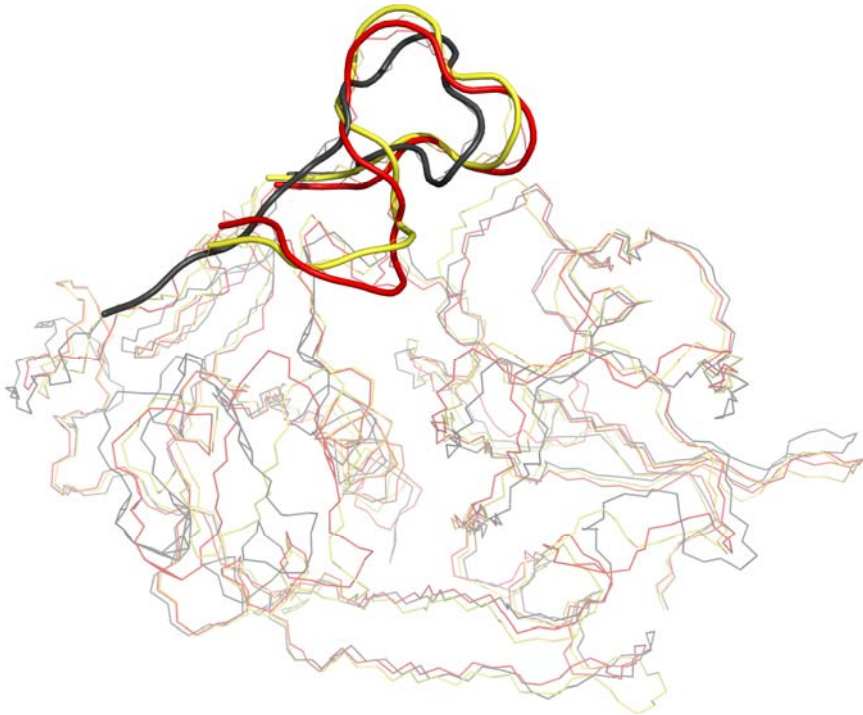

— *apo* / pH 8

— + hep (R-domain) / pH 8

— + hep (L-domain) / pH 8

Supplement: Additional file 4 — Heparin binding induces a distinct conformation of the occluding loop Structural alignment of the average structures obtained from the MD simulations in alkaline conditions. Color definitions are represented. We represented the occluding loop region in tubes to highlight the distinct conformation adopted by this structural element when catB binds heparin. [file 1471-2164-11-S5-S5-S4.pdf]
